# Proteasomes Are Critical for Maintenance of CD133+CD24+ Kidney Progenitor Cells

**DOI:** 10.3390/ijms241713303

**Published:** 2023-08-27

**Authors:** Sarmad Al-Marsoummi, Aaron A. Mehus, Swojani Shrestha, Rayna Rice, Brooke Rossow, Seema Somji, Scott H. Garrett, Donald A. Sens

**Affiliations:** Department of Pathology, School of Medicine and Health Sciences, University of North Dakota, Grand Forks, ND 58202, USA

**Keywords:** HRTPT, kidney progenitor, proteasomes, HREC24T, proximal tubule, proteasomal inhibitors

## Abstract

Kidney progenitor cells, although rare and dispersed, play a key role in the repair of renal tubules after acute kidney damage. However, understanding these cells has been challenging due to the limited access to primary renal tissues and the absence of immortalized cells to model kidney progenitors. Previously, our laboratory utilized the renal proximal tubular epithelial cell line, RPTEC/TERT1, and the flow cytometry technique to sort and establish a kidney progenitor cell model called Human Renal Tubular Precursor TERT (HRTPT) which expresses CD133 and CD24 and exhibits the characteristics of kidney progenitors, such as self-renewal capacity and multi-potential differentiation. In addition, a separate cell line was established, named Human Renal Epithelial Cell 24 TERT (HREC24T), which lacks CD133 expression and shows no progenitor features. To further characterize HRTPT CD133+CD24+ progenitor cells, we performed proteomic profiling which showed high proteasomal expression in HRTPT kidney progenitor cells. RT-qPCR, Western blot, and flow cytometry analysis showed that HRTPT cells possess higher proteasomal expression and activity compared to HREC24T non-progenitor cells. Importantly, inhibition of the proteasomes with bortezomib reduced the expression of progenitor markers and obliterated the potential for self-renewal and differentiation of HRTPT progenitor cells. In conclusion, proteasomes are critical in preserving progenitor markers expression and self-renewal capacity in HRTPT kidney progenitors.

## 1. Introduction

The inherent capacity of human kidneys for regeneration and repair after acute injury is an intriguing phenomenon that has been a research focus for many years [1]. Central to this regenerative process is a rare but unique population of kidney progenitor cells that have the capacity to differentiate into multiple renal lineages and are responsible for kidney repair, and can potentially offer therapeutic solutions to renal disease [2,3]. Among these populations of renal progenitor cells, CD133+CD24+ kidney progenitor cells have emerged as a significant player in nephrogenesis and renal regeneration after damage [4,5]. CD133+CD24+ kidney cells have been identified in human fetal and adult kidneys, suggesting their persistent role in kidney homeostasis and repair beyond fetal development [6]. It has been established that kidney progenitors that repair tubular damage after injury, including CD133+CD24+ progenitor cells, reside within the kidney and are not recruited from outside the kidney [7]. 

CD133+CD24+ kidney progenitors were first isolated and successfully derived from primary human kidney cortical renal epithelial cultures [4,8,9]. To understand how the kidney repairs itself, we need to characterize and understand kidney progenitor cells. Kidney progenitors are a rare, scattered cell population, and kidney stem cell research lagged and remained in its infancy due to the limited supply and access of primary cultures, and hence kidney progenitors, and the absence of immortalized kidney progenitor cell model.

Previously, this lab demonstrated that both the immortalized proximal tubular cell model RPTEC/TERT1 and human primary cortical tissue cells contained a subset of cells co-expressing CD133 and CD24, as well as another population of cells that expressed only CD24 [10], and successfully isolated these two cell populations and established two new cell lines that are able to grow in culture and maintain their phenotype [11]. The first cell line was identified as Human Renal Tubular Precursor TERT (HRTPT), which expresses both CD133 and CD24 and shows most of the key characteristics of stem/progenitor cells, including self-renewal and multipotent differentiation capacity [11], and shares 332 co-expressed genes with infant CD133+ kidney progenitor cells [12]. On the contrary, the other cell line, identified as Human Renal Epithelial Cell 24 TERT (HREC24T), expresses only CD24 and shows no stem/progenitor characteristics [11]. Therefore, our HRTPT cells were established to serve as an immortalized kidney progenitor cell model and provide unlimited supply for kidney stem/progenitor studies. 

Proteasomes are protein complexes that are critical for maintaining cell functions and controlling protein turnover inside the cell [13]. Proteasomes have been implicated as critical to maintaining pluripotency in embryonic stem cells [14,15,16,17,18] and in differentiating neural progenitor cells [19]. However, no data are available about the role of proteasomes in any of the identified kidney progenitors, including CD133+CD24+, and there is scarce data about the role of proteasomes in kidney diseases. 

The aim of this study was to further characterize HRTPT CD133+CD24+ kidney progenitor cells and how they are different from the non-progenitor cells to identify the cellular and molecular signals that are critical for the maintenance and function of HRTPT kidney progenitor cells, and to determine the role of proteasomes in HRTPT CD133+CD24+ kidney progenitor cells.

## 2. Results

### 2.1. Proteasomal Profile of HRTPT CD133+/CD24+ Kidney Progenitor

**Figure 1:**
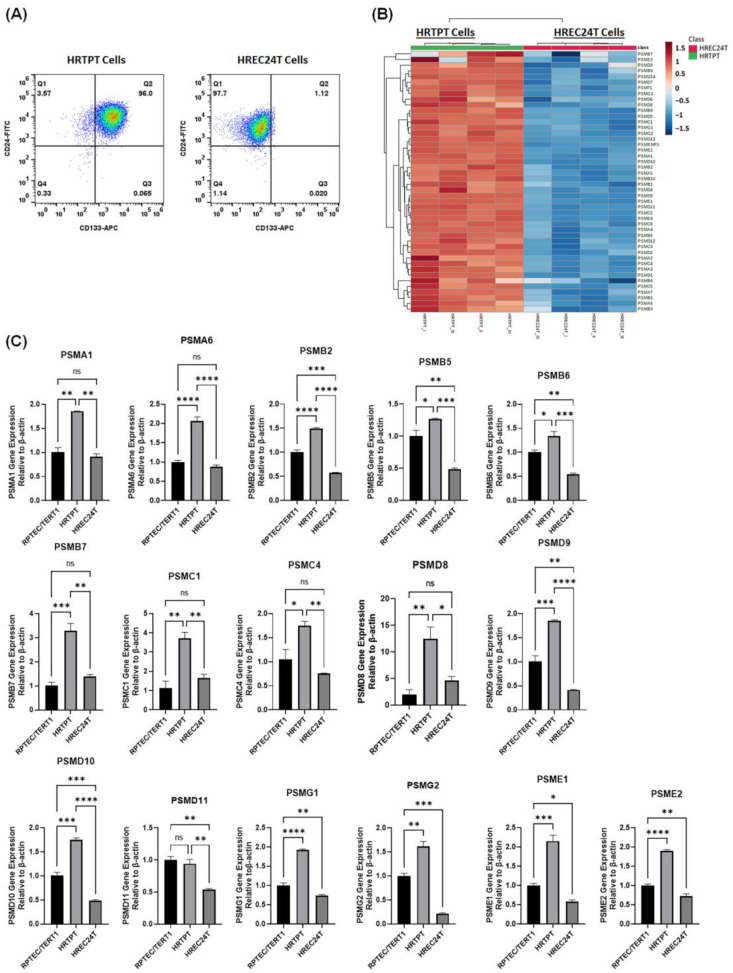
HRTPT CD133+CD24+ kidney progenitors are characterized by increased proteasomal subunit expression. (**A**) Flow cytometry analysis of CD133 and CD24 expression in HRTPT and HREC24T cell lines showing HRTPT cells express both CD133 and CD24, while HREC24T cells lack CD133 expression. (**B**) Heat map of proteomic profile analysis of four independent isolates of HRTPT kidney progenitors (HRTPT I-IV) versus HREC24T non-progenitors (HREC24T I-IV) demonstrates upregulation of the 46 proteasomal proteins in HRTPT cells compared to HREC24T. (**C**) mRNA levels analysis by RT-qPCR demonstrates a significant increase in proteasomal subunits (*PSMA1, PSMA6, PSMB2, PSMB5, PSMB6, PSMB7, PSMC1, PSMC4, PSMD8, PSMD9, PSMD10, PSMD11, PSMG1, PSMG2, PSME1, PSME2*) in HRTPT cells versus HREC24T cells (*n* = 3, one-way ANOVA, ns: not significant, * *p* < 0.05, ** *p* < 0.01, *** *p* < 0.001, **** *p* < 0.0001). All data are expressed as mean ± SEM.

Two distinct cell line models, which were derived from the RPTEC-TERT1 proximal tubular cell line, were previously established and characterized in our laboratory [11]. One cell line expressed both CD133 and CD24 and exhibited progenitor features; hence, it was named Human Renal Tubular Precursor TERT (HRTPT). The other cell line expressed only CD24 and lacked CD133 expression and the progenitor characteristics, which was denoted Human Renal Epithelial Cell 24TERT (HREC24T). These two cell lines maintained their phenotype and expression pattern of CD133 and CD24 over a series of passages (Figure 1A). In order to better understand the differences between the kidney progenitor and non-progenitor cells, proteomic profiling was performed on both HRTPT (CD133+CD24+) kidney progenitor cells and HREC24T (CD24+) non-progenitor cells. Proteomic analysis revealed significantly higher expression of proteasomal proteins in HRTPT kidney progenitor cells compared to the non-progenitor HREC24T cells that do not express CD133 (Figure 1B).

Further validation by analyzing mRNA levels using RT-qPCR demonstrated a significant increase in the expression of proteasomal genes in HRTPT progenitor cells relative to non-progenitor HREC24T cells. Remarkably, the expression levels of selected proteasomal genes (*PSMA1, PSMA6, PSMB2, PSMB5, PSMB6, PSMB7, PSMC1, PSMC4, PSMD8, PSMD9, PSMD10, PSMD11, PSMG1, PSMG2, PSME1, PSME2*) were significantly increased in HRTPT kidney progenitor cells relative to HREC24T non-progenitor cells (Figure 1C).

### 2.2. HRTPT CD133+CD24+ Kidney Progenitor Cells Are Characterized by Increased Proteasomal Proteins and Catalytic Activity

**Figure 2:**
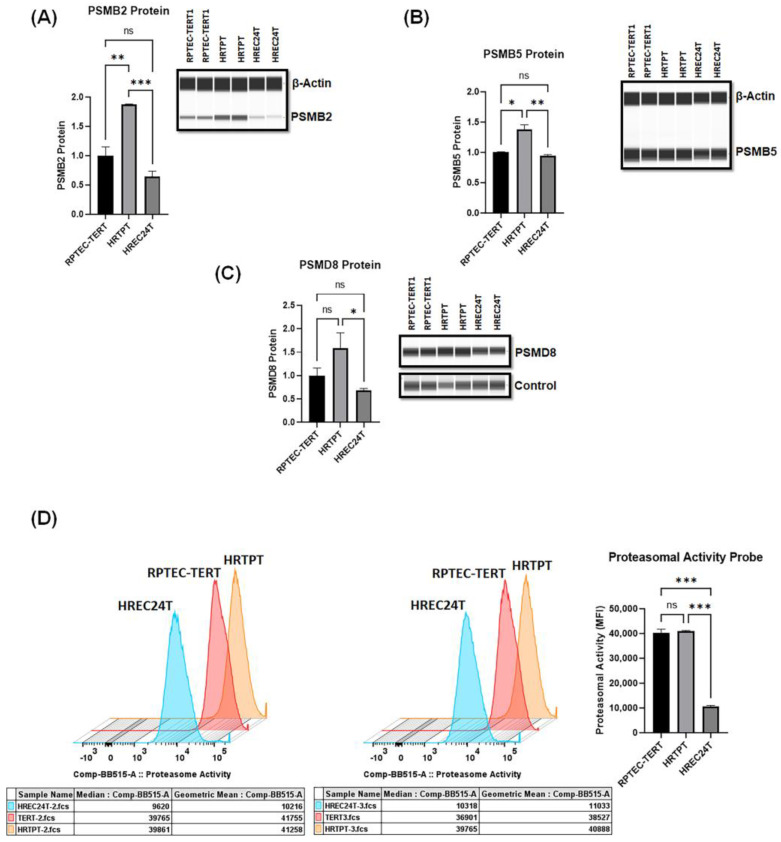
HRTPT kidney progenitor cells are characterized by increased expression of proteasomal proteins and proteasomal catalytic activity. (**A**−**C**) Protein levels analysis by ProteinSimple demonstrated a significant increase in the levels of proteasomal proteins (PSMB2, PSMB5, and PSMD8) in HRTPT kidney progenitors versus HERC24T non-progenitor cells and parent proximal tubular cells (RPTEC−TERT1). (**D**) Flow cytometry analysis using a fluorescent proteasomal activity probe (Me4BodipyFL-Ahx3Leu3VS); HRTPT, HREC24T, and RPTEC-TERT cells were cultured with 5 µM of the probe for 6 h before processed for flow cytometry. The analysis demonstrated a significantly increased proteasomal activity of HRTPT progenitor cells relative to HREC24T cells. (*n* = 3, one-way ANOVA, ns: not significant, * *p* < 0.05, ** *p* < 0.01,*** *p* < 0.001). All data are expressed as mean ± SEM.

To confirm and validate our previous proteomic and transcriptomic data, we utilized ProteinSimple analysis to verify that HRTPT (CD133+CD24+) kidney progenitor cells have higher protein levels of selected proteasomal subunits, including catalytic activity core subunits (PSMB2 and PSMB5). HRTPT (CD133+CD24+) kidney progenitor cells showed significantly higher levels of PSMB2, PSMB5, and PSMD8 proteasomal proteins compared to HREC24T non-progenitor cells (Figure 2A–C).

Next, we sought to determine whether the higher expression of proteasomal proteins in HRTPT (CD133+CD24+) kidney progenitor cells is correlated with increased proteasomal catalytic activity in HRTPT kidney progenitor cells compared to HREC24T non-progenitors. Flow cytometry analysis using a proteasomal activity probe demonstrated that proteasomal catalytic activity was significantly increased in HRTPT kidney progenitors compared to HREC24T non-progenitor cells (Figure 2D). However, proteasomal activity levels in HRTPT cells were comparable to those of RPTEC-TERT parent cells (Figure 2D).

### 2.3. Effect of Proteasomal Inhibition on Morphology and Viability of HRTPT Kidney Progenitor Cells

**Figure 3:**
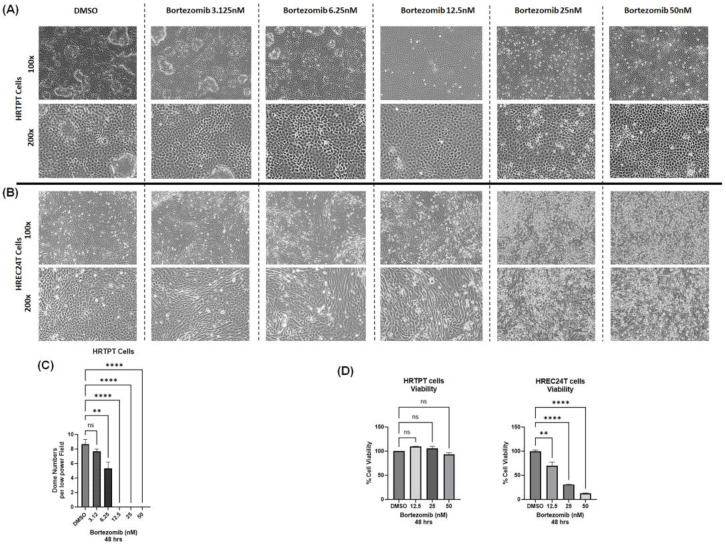
Proteasomal inhibition reduces dome formation with minimal cytotoxicity in HRTPT progenitor cells. Light microscopy images (100× and 200× magnification) showing the effect of 48 h treatment with bortezomib (3.125, 6.25, 12.5, 25, 50 nM) in (**A**) HRTPT progenitor cells, and (**B**) HREC24T progenitor cells. Bortezomib reduced dome formation in HRTPT cells at 12.5 nM, 25 nM, and 50 nM with minimal signs of cytotoxicity. HREC24T non-progenitor cells showed signs of cytotoxicity due to bortezomib treatment at 12.5 nM, 25 nM, and 50 nM. (**C**) Numbers of domes counted per low power field (10× objective) in HRTPT cells treated with bortezomib for 48 h in three independent experiments. (**D**) Cell viability assay by crystal violet labeling and absorbance in HRTPT and HREC24T cells showing bortezomib cytotoxicity and reduced cell numbers in HREC24T but not HRETPT cells. (*n* = 2–3, one-way ANOVA, ns: not significant ** *p* < 0.01, **** *p* < 0.0001). All data are expressed as mean ± SEM.

Having demonstrated that HRTPT (CD133+CD24+) kidney progenitor cells are characterized by high proteasomal catalytic activity, we next wanted to identify the effect of inhibiting the proteasomal activity in HRTPT kidney progenitors. Inhibiting the proteasomes in HRTPT kidney progenitor cells by treatment with 3.12, 6.25, 12.5, 25, and 50 nM of bortezomib for 48 h caused a dose-dependent reduction in dome formation (feature of vectorial active transport) at 12.5 nM or higher concentration (Figure 3A); nevertheless, HRTPT progenitor cells exhibited minimal signs of cytotoxicity due to bortezomib treatment even at higher doses (Figure 3A,D). In addition, there was no upregulation of kidney cell injury markers (*KIM1, KRT8, KRT18, KRT19*) (Figure S2). On the contrary, treating HREC24T non-progenitor cells with bortezomib, at a dose of more than 12.5 nM, elicited clear signs of cytotoxicity, suggesting increased sensitivity of HREC24T non-progenitor kidney cells to proteasomal inhibition compared to HRTPT kidney progenitor cells (Figure 3B,D).

### 2.4. Proteasomal Inhibition Reduces the Expression of CD133 Progenitor Marker in HRTPT CD133+CD24+ Progenitor Cells

**Figure 4:**
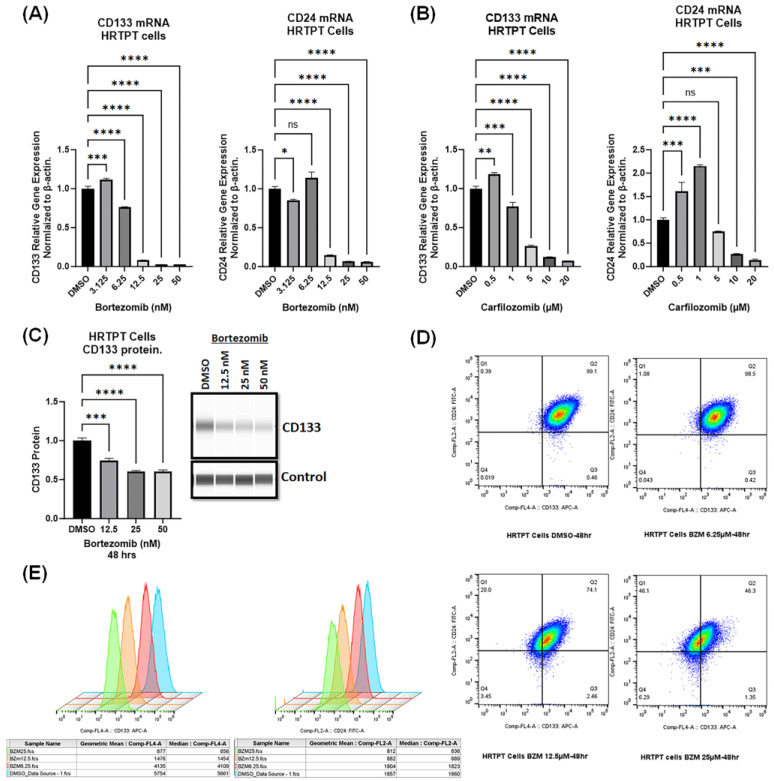
Proteasomal inhibition reduces the expression of CD133 and CD24 in HRTPT kidney progenitor cells. CD133 and CD24 mRNA levels analysis via RT-qPCR in HRTPT progenitor cells that were treated for 48 h with (**A**) bortezomib (3.12, 6.25, 12.5, 25, 50 nM) and (**B**) carfilzomib (0.5, 1, 5, 10, 20 µM). (**C**) CD133 protein levels analysis via ProteinSimple in HRTPT progenitor cells treated with bortezomib 12.5 nM, 25 nM, and 50 nM for 48 h. (**D**,**E**) Flow cytometry analysis of CD133 and CD24 in HRTPT progenitor cells treated with DMSO (blue color) or bortezomib (BZM) (6.25 nM (red color), 12.5 nM (orange color), and 25 nM (green color)) for 48 h. (B-actin and ProteinSimple system control were used as a reference gene and reference protein, respectively. (*n* = 3, one-way ANOVA, ns: not significant, * *p* < 0.05, ** *p* < 0.01, *** *p* < 0.001, **** *p* < 0.0001). All data are expressed as mean ± SEM.

Given that HRTPT cells demonstrated increased proteasomal activity, we aimed to determine whether the elevated proteasomal activity in HRTPT cells is critical for the expression of the progenitor marker (CD133) that defines these cells. Inhibition of proteasomal activity in HRTPT cells with 6.25, 12.5, 25, and 50 nM of bortezomib for 48 h resulted in a dose-dependent and significant reduction in mRNA levels of the stem/progenitor markers CD133 and CD24 (Figure 4A). Bortezomib as low as 6.25 nM significantly reduced CD133 mRNA levels, but the reduction in CD24 mRNA levels was observed at bortezomib concentration of 12.5 nM; therefore, we chose a 12.5 nM concentration of bortezomib for further analysis. Furthermore, bortezomib caused a dose-dependent reduction in other progenitor markers (*ALDH1A1* and *PAX2*) (Figure S2).

To rule out an idiosyncratic effect of bortezomib, we used carfilzomib, which is another clinically approved proteasomal inhibitor. Analogous to the effects of bortezomib, carfilzomib decreased the expression of both CD133 and CD24 in HRTPT cells that were treated with 5, 10, and 20 µM of carfilzomib (Figure 4B). A quantity of 1 µM of carfilzomib caused a significant reduction in CD133 mRNA levels, but not in those of CD24. Light microscopy images of carfilzomib treatment are available in the Supplementary Data (Figure S3).

ProteinSimple confirmed that CD133 protein levels were significantly reduced in HRTPT cells treated with 12.5, 25, or 50 nM bortezomib for 48 h (Figure 4C). Additionally, flow cytometry analysis of HRTPT progenitor cells showed a reduction in the CD133+CD24+ cell population 48 h after 12.5 nM or 50 nM bortezomib treatment (Figure 4D), in addition to a reduction in the surface expression levels of CD133 and CD24 in HRTPT cells (Figure 4E).

### 2.5. Prolonged Bortezomib Cyclic Regimen Alters the Morphology and Expression of Progenitor Markers in HRTPT Progenitor Cells

**Figure 5:**
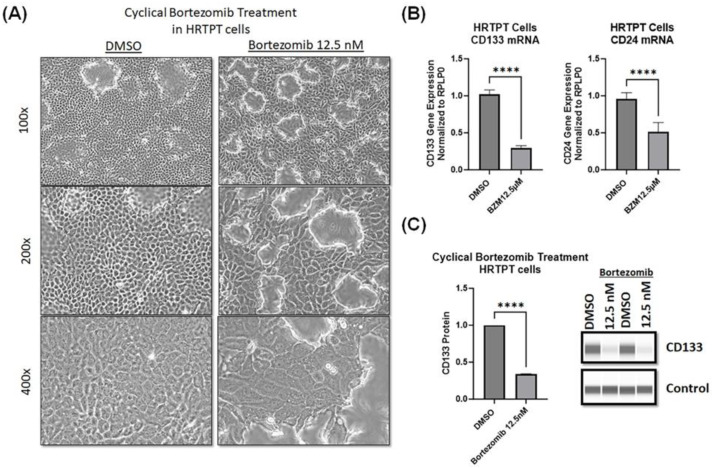
Cyclical treatment with bortezomib alters the morphology of HRTPT progenitor cells. (**A**) Light microscopy showing the morphology of HRTPT progenitor cells at the end of the treatment cycle of bortezomib (12th day); in brief, 12.5 nM bortezomib was added on 1st day, 4th day, 8th day, and 11th day for 24 h each time, then cells were replaced with drug-free medium until the next dose. (**B**) RT-qPCR analysis of mRNA levels of CD133 and CD24 in HRTPT progenitor cells at the end of bortezomib treatment cycle (day 12), (*n* = 3, RPLP0 used as a reference gene, Student’s *t*-test, **** *p* < 0.0001). (**C**) ProteinSimple analysis of CD133 protein levels in HRTPT progenitor cells at the end of bortezomib treatment cycle (day 12), (*n* = 2, ProteinSimple system control protein used as a reference normalization protein, Student’s *t*-test, **** *p* < 0.0001).

Since bortezomib is a drug that is used clinically to treat patients with multiple myeloma and is administered in a cycle regimen, we sought to determine if such a cycle treatment regimen will affect the HRTPT progenitor cells. Interestingly, cyclical treatment of HRTPT progenitor cells with 12.5 nM bortezomib that was added on the 1st day, 4th day, 8th day, and 11th day caused a morphology alteration in HRTPT progenitor cells evident as cellular hypertrophy, loss of dome formation, and visible double nucleoli, but no signs of cytotoxicity were observed (Figure 5). The analysis of mRNA and protein levels demonstrated that these morphological changes in the HRTPT progenitor cells were associated with a significant reduction in the expression of the progenitor marker CD133 at both mRNA and protein levels (Figure 5).

### 2.6. Proteasomal Inhibition Abolishes Sphere Formation Ability of HRTPT Kidney Progenitor Cells

**Figure 6:**
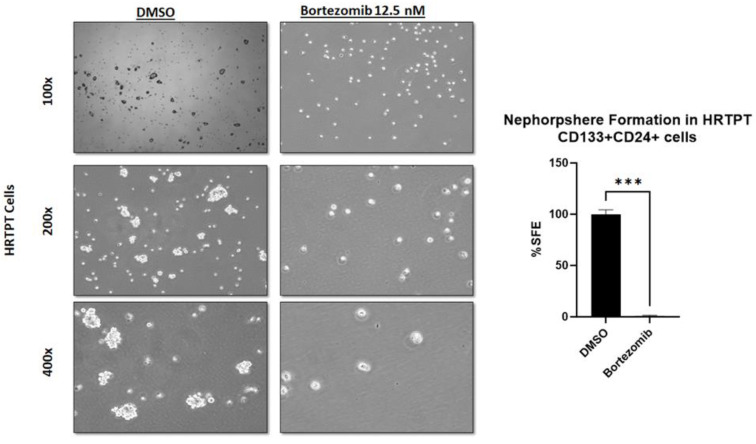
Proteasomal inhibition abolishes the sphere formation ability of HRTPT kidney progenitor cells. Light microscopy images of spheres formation in HRTPT cells. Pretreating HRTPT cells with 12.5 nM bortezomib, 48 h before seeding for the sphere assay, significantly reduced the sphere formation efficiency (%SFE). %SFE was calculated by the formula (number of spheres/number of the seeded cellsx100), (*n* = 3, Student’s *t*-test, *** *p* < 0.001). All data are expressed as mean ± SEM.

We have previously shown that HRTPT CD133+CD24+ kidney progenitor cells are characterized by the ability to form spheres as functional evidence of self-renewal [11]. We intended to determine if proteasomal inhibition will affect the sphere formation ability of the HRTPT kidney progenitor cells. Pretreatment of HRTPT cells with 12.5 nM bortezomib for 48 h before seeding for sphere formation significantly reduced the sphere formation ability of HRTPT CD133+CD24+ kidney progenitors (Figure 6), indicating that proteasomal inhibition not only reduced the expression of progenitor markers, but also reduced self-renewal ability of the HRTPT progenitor cells, which is a hallmark feature of stem/progenitor cells.

### 2.7. Proteasomal Inhibition Impairs Tubular Differentiation of Kidney Progenitors

**Figure 7:**
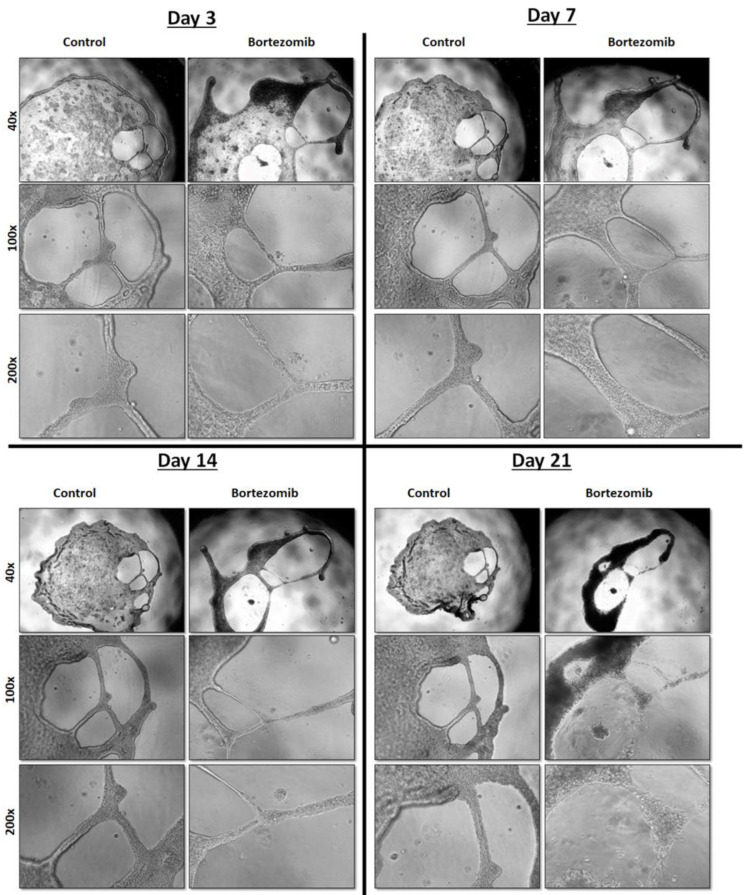
Proteasomal inhibition by bortezomib impaired the tubular differentiation of HRTPT cells. Light microscopy images (40×, 100×, and 200× magnification) of HRTPT progenitor cells that were grown on the surface of a Matrigel™ coated 96-well plate, in presence of bortezomib 12.5 nM or DMSO as a control, showing the tubular-like structures disintegrated by day 21 in presence of 12.5 nM bortezomib.

We have previously shown that HRTPT CD133+CD24+ progenitor cells are characterized by their ability to differentiate into tubular-like structures when grown on the surface of Matrigel™ [11], as a characteristic feature of kidney progenitor cells. Therefore, we wanted to investigate if proteasomal inhibition will affect the tubular differentiation ability of HRTPT cells. Culturing HRTPT on Matrigel™ in the presence of 12.5 nM bortezomib did not block the initial formation of tubular-like structures (Figure 7). However, the long-term growth and support of these tubular-like structures were decreased, and the walls of the tubular structures disintegrated on day 21 (Figure 7) in the presence of bortezomib compared to control cells treated with DMSO, indicating the ability of HRTPT cells to maintain and support the tubular-like differentiation was reduced due to proteasomal inhibition by bortezomib.

**Figure 8:**
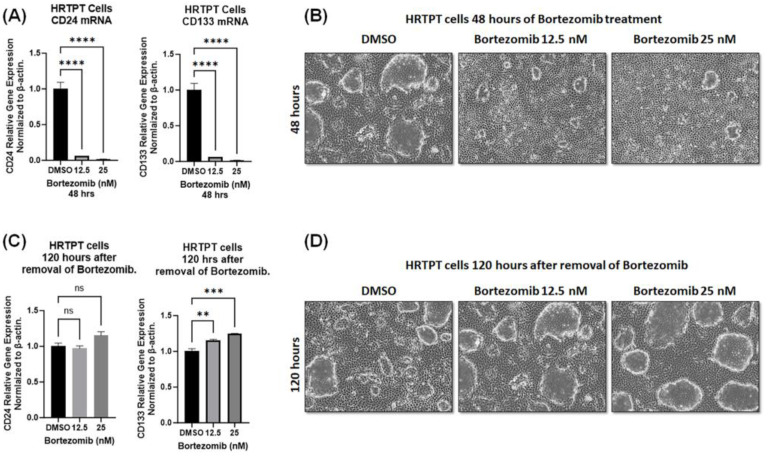
HRTPT cells can restore CD133 expression after short-term proteasomal inhibition. HRTPT cells treated with 12.5 nM and 25 nM bortezomib for 48 h showing (**A**) significantly reduced mRNA levels of CD133 and CD24 and (**B**) reduced dome formation when observed under light microscopy. Removal of bortezomib and culturing cells in bortezomib-free media for 120 h caused (**C**) re-expression of CD133 and CD24 and (**D**) restoration of the dome-formation ability. (*n* = 3, one-way ANOVA, ns: not significant, ** *p* < 0.01, *** *p* < 0.001, **** *p* < 0.0001). All data are expressed as mean ± SEM.

Interestingly, HRTPT cells restored normal morphology and CD133 expression after the removal of bortezomib (Figure 8).

## 3. Discussion

The present study aimed to further characterize the immortalized CD133+CD24+ Human Renal Tubular Precursor TERT (HRTPT) cells that were previously established in our laboratory [11], and to investigate the role of proteasomes in these cells.

Our results demonstrate several important findings that contribute to our understanding of CD133+ CD24+ kidney progenitor cells and the importance of proteasomal activity in these cells.

The identification and characterization of kidney progenitor cells have been of great interest in the field of regenerative medicine. Kidney progenitors are rare and scattered populations of cells. In 2005, Bussolati et al. discovered adult human kidney-derived CD133+ progenitor cells that also expressed Pax2, which is an embryonic kidney marker, and were capable of in vitro expansion and self-renewal [4]. Later, others revealed that CD133+CD24+ kidney progenitor cells are capable of clonal expansion and repair of damage done to the tubular cells, making them a significant player in nephrogenesis and renal regeneration after injury [8,9,20,21,22,23]. Since their discovery, the source for these cells remained the primary cortical kidney cultures [4,8,23,24]. However, using primary cultures as a research model presents significant challenges due to difficulties in securing human cortical tissue, and the high level of expertise required for isolation and cultivation of primary cells. The immortalized renal proximal tubular epithelial (RPTEC/TERT1) cell line has emerged as a particularly promising and manageable culture system for modeling the cellular aspect of the renal tubular epithelium [25]. An intriguing observation by this laboratory was that approximately 80% of the RPTEC/TERT1 cells concurrently express the CD133 and CD24 markers [10], which was comparable to human primary cortical tissue cells [10]. This finding lends considerable weight to the argument that the RPTEC-TERT1 cell line could be an invaluable resource in elucidating the intricate mechanisms that underpin the ability of these cells to regenerate tubular epithelium. On the other hand, the distinct 20% of RPTEC/TERT1 cells that do not express CD133 and only express CD24 could potentially be progenitor cells that differentiated towards another cell lineage. Nevertheless, these CD24 cells present us with the opportunity of a background cell or control cell to study kidney progenitors, thereby further diversifying the potential uses and understanding of these cell populations. Based on these observations, in 2017, this laboratory successfully sorted and established two cell lines from RPTEC-TERT1 [10,11]; the first cell line, identified as Human Renal Tubular Precursor TERT (HRTPT), which co-expresses CD133 and CD24, and another cell line identified as Human Renal Epithelial Cell 24TERT (HREC24T), which only expresses CD24 [11]. In addition to expressing CD133 and CD24, HRTPT cells show multipotential differentiation capacity, and the ability to form spheres and tubule-like structures when grown on the surface of Matrigel™ [11]. Remarkably, HRTPT cells share 332 co-expressed genes with the infant CD133+ cells and 151 co-expressed genes with the urinary-derived human renal progenitor cells [26]. 

Collectively, the previous evidence supports using HRTPT cells as an immortalized cell model of kidney progenitors, and importantly provides the opportunity to compare these cells to HREC24T kidney cells that are derived from the same tissue, but lack the expression of CD133, and do not show any progenitor features.

To better characterize HRTPT progenitor cells and understand the molecular signals that are critical for their progenitor status and are important for kidney repair after damage, we performed proteomic analysis on both the HRTPT and the HREC24T cells. Our data showed that the HRTPT cells expressed high levels of proteasomal proteins compared to the non-progenitor cells (HREC24T). This finding was validated on both RNA and protein levels in addition to the determination of a higher proteasomal catalytic activity in HRTPT progenitor cells compared to HREC24T non-progenitor cells. This finding is intriguing as the two cell populations were originally mixed together in the parent RPTEC-TERT1 cells, indicating that the RPTEC-TERT1 cells are not only a heterogenous population of cells that differ in the expression of cell surface markers, but also a heterogenous population of cells that differ in protein homeostasis. Proteasomes are essential protein complexes involved in maintaining cellular functions and regulating protein turnover. Previous studies have implicated the role of proteasomes in maintaining pluripotency in embryonic stem cells [12,13,14,15,16], in the differentiation of neural progenitor cells [17], and in mesenchymal stem cell differentiation [27]. However, the role of proteasomes in kidney stem/ progenitor cells and kidney diseases remains poorly explored. Although no data are available regarding the proteasomal expression in kidney progenitors, our analysis of publicly available datasets revealed that there is an upregulation of the expression of the majority of proteasomal genes in nephrogenic lineages in 17-week human fetal kidney [28,29,30], indicating the need for high proteasomal activity during nephrogenesis when kidney progenitors are highly active. 

The notion that our HRTPT cells demonstrated an increased proteasomal expression activity, which is a feature that is shared with embryonic stem cells and neuronal progenitor cells, further supports the argument that HRTPT cells are progenitor cells and can be used as an immortalized cell model of kidney progenitor cells. However, this finding does raise the question of whether the reduction in proteasomal activity in HRTPT cells affects the progenitor characteristics and differentiation capacity of these cells. 

Our data not only showed that HRTPT cells are characterized by higher proteasomal expression and activity, but also demonstrated that blocking proteasomal catalytic activity caused a loss in CD133 expression, which is a critical progenitor marker in the kidney [4,6,9,21,31,32], and abolished the sphere formation ability of HRTPT cells, indicating the loss of self-renewal ability that defines these progenitor cells. One fundamental feature of stem and progenitor cells is their ability to balance self-renewal and differentiation. Several studies have implicated the proteasome in regulating this delicate equilibrium in stem cells [14,15,16,33,34]. Human embryonic stem cells (hESCs) and induced pluripotent stem cells have been shown to be characterized by increased proteasomal activity relative to their differentiated cells [18]. For instance, inhibition of proteasome activity in embryonic stem cells (ESCs) leads to impaired self-renewal and increased differentiation [14], suggesting that proteasomal degradation is vital for maintaining the pluripotent state [16]. Additionally, the proteasome has been implicated in the degradation of specific transcription factors and regulators, such as Oct4 and Nanog, which are critical for maintaining the undifferentiated state of ESCs [33,34]. The previous studies collectively identified the role of the proteasomes in orchestrating the balance between self-renewal and differentiation in stem and progenitor cells, and support our data that demonstrated inhibiting the proteasomal activity caused the loss of the features that define HRTPT cells as kidney progenitors, namely CD133 expression and sphere formation ability (indicative of self-renewal). Additionally, proteasomal inhibition reduced the expression of PAX2, ALDH1A1, and OCT4, which are all stem markers that are required to maintain progenitor status. 

Our results showed a contrasting effect of proteasomal inhibition on cell viability of HRTPT versus HREC24T cells. HRTPT cells were more resistant to proteasomal inhibition with minimal cytotoxic effect, even with a high concentration of bortezomib. In fact, markers of kidney tubular cell injury (KIM1, KRT8, KRT18, KRT19) [35,36] were reduced with proteasomal inhibition by bortezomib in HRTPT cells. In contrast, HREC24T cells showed signs of cytotoxicity due to proteasomal inhibition with bortezomib, even at low concentrations. This indicates that HREC24T cells lowered their proteasomal catalytic activity to the level that is needed to sustain the function of the cells, and any further reduction would have a detrimental effect on cell function and, hence, cell survival. Therefore, a reduction in proteasomal activity causes a more detrimental effect on the cell survival of HREC24T cells compared to the HRTPT progenitor cells that have higher proteasomal activity. 

Our data showed that the effect of short-term proteasomal inhibition was reversible and that HRTPT cells restored the expression of the progenitor marker CD133, indicating the dynamic nature of these cells that requires rapid adjustment during injury to regenerate tubular cells and, at the same time, maintain their existence. Although not within the scope of this study, it would be interesting to determine the transcriptomic and proteomic differences during proteasomal inhibition to determine the pathways that are critical in progenitor cell differentiation and maintenance of their pool. 

Interestingly, prolonged cyclical treatment with bortezomib, to mimic the treatment regimen used in multiple myeloma patients, led to morphological alterations in HRTPT progenitor cells, including cellular hypertrophy, loss of dome formation, and visible double nucleoli with no signs of cytotoxicity. Similar to short-term proteasomal inhibition, these changes were accompanied by a significant reduction in CD133 expression at both mRNA and protein levels. This indicates that the use of proteasomal inhibitors in patients with multiple myeloma might impact the self-renewal capacity of kidney progenitor cells and might deplete these cells, which causes a future reduced potential to recover from acute kidney damage. 

Although proteasomal inhibition blocked the sphere formation in HRTPT cells, indicating the loss of self-renewal ability of these cells, it did not block the initial development of the tubular-like structures when these cells were grown on the surface of Matrigel™. Nonetheless, it diminished the support of the tubular growth as the cells started to disintegrate by day 21 in the presence of bortezomib, indicating the loss of progenitor function which is required to maintain and support the established tubules. 

In conclusion, our results demonstrate the importance of proteasomal activity in the maintenance of HRTPT (CD133+CD24+) kidney progenitor cells. Proteasomal inhibition affected their morphology, expression of progenitor markers, self-renewal ability, and tubular differentiation potential. These findings highlight the significance of proteasomal regulation in the functional properties of kidney progenitor cells and provide insights into the potential therapeutic implications of targeting proteasomes in renal regeneration and disease. While our study sheds light on the role of proteasomes in HRTPT kidney progenitor cells, further investigations, such as single-cell RNAseq of RPTEC-TERT cells to better understand the heterogeneity of these cells and how the heterogenous cell populations behave when they are mixed and cross talk, are warranted to fully elucidate the underlying molecular mechanisms. It would be valuable to explore the specific proteasomal subunits and pathways involved in regulating the functions of kidney progenitor cells. Additionally, the interaction between proteasomes and other signaling pathways implicated in kidney development and regeneration should be investigated to better understand the complex regulatory networks at play.

## 4. Materials and Methods

### 4.1. Cell Culture and Reagents

RPTEC-TERT cells were acquired from American Type Culture Collection (ATCC); HRTPT and HREC24T cell lines were previously established in this laboratory [10,11]. All cell lines were cultured in serum-free condition using a 1:1 mixture of DMEM: F12 serum-free media supplemented with selenium (5 ng/mL), insulin (5 μg/mL), transferrin (5 μg/mL), hydrocortisone (36 ng/mL), triiodothyronine (4 pg/mL), and epidermal growth factor (10 ng/mL). Cells were cultured at 37 °C and 5% CO_2_ and fed fresh medium every three days, and upon confluency, cells were passaged at a 1:3 ratio. Bortezomib and carfilzomib were purchased from Selleckchem (#S1013, #S2853, Houston, TX, USA).

### 4.2. Proteomics Analysis (CME bHPLC TMT Methods—Orbitrap Eclipse)

Total protein from cultured cell pellets was reduced, alkylated, and purified by chloroform/methanol extraction prior to digestion with sequencing grade trypsin and LysC (Promega). The resulting peptides were labeled using a tandem mass tag 10-plex isobaric label reagent set (Thermo Fisher Scientific), then enriched using High-Select TiO_2_ and Fe-NTA phosphopeptide enrichment kits (Thermo Fisher Scientific, Waltham, MA, USA) following the manufacturer’s instructions. Both enriched and un-enriched labeled peptides were separated into 46 fractions on a 100 × 1.0 mm Acquity BEH C18 column (Waters Corporation, Milford, MA, USA) using an UltiMate 3000 UHPLC system (Thermo Fisher Scientific) with a 50 min gradient from 99:1 to 60:40 buffer A:B ratio under basic pH conditions, then consolidated into 18 super-fractions. Each super-fraction was then further separated by reverse phase XSelect CSH C18 2.5 um resin (Waters) on an in-line 150 × 0.075 mm column using an UltiMate 3000 RSLCnano system (Thermo Fisher Scientific). Peptides were eluted using a 75 min gradient from 98:2 to 60:40 buffer A:B ratio. Eluted peptides were ionized by electrospray (2.4 kV) followed by mass spectrometric analysis on an Orbitrap Eclipse Tribrid mass spectrometer (Thermo Fisher Scientific) using multi-notch MS3 parameters. MS data were acquired using the FTMS analyzer in top-speed profile mode at a resolution of 120,000 over a range of 375 to 1500 *m*/*z*. Following CID activation with normalized collision energy of 31.0, MS/MS data were acquired using the ion trap analyzer in centroid mode and normal mass range. Using synchronous precursor selection, up to 10 MS/MS precursors were selected for HCD activation with normalized collision energy of 55.0, followed by acquisition of MS3 reporter ion data using the FTMS analyzer in profile mode at a resolution of 50,000 over a range of 100–500 *m*/*z*. Buffer A = 0.1% formic acid, 0.5% acetonitrile, Buffer B = 0.1% formic acid, 99.9% acetonitrile. Both buffers were adjusted to pH 10 with ammonium hydroxide for offline separation. Data Analysis—ProteoViz (phosphoTMT): Proteins were identified and reporter ions quantified by searching the UniprotKB database restricted to Homo sapiens (June 2021) using MaxQuant (Max Planck Institute, version 2.0.3.0) with a parent ion tolerance of 3 ppm, a fragment ion tolerance of 0.5 Da, a reporter ion tolerance of 0.001 Da, trypsin/P enzyme with 2 missed cleavages, variable modifications including oxidation on M, Acetyl on Protein N-term, and phosphorylation on STY, and fixed modification of Carbamidomethyl on C. Protein identifications were accepted if they could be established with less than 1.0% false discovery. Proteins identified only by modified peptides were removed. Protein probabilities were assigned by the Protein Prophet algorithm [37]. TMT MS3 reporter ion intensity values are analyzed for changes in total protein using the unenriched lysate sample. Phospho(STY) modifications were identified using samples enriched for phosphorylated peptides. The enriched and un-enriched samples were multiplexed using two TMT10-plex batches, one for the enriched and one for the un-enriched samples.

Following data acquisition and database search, the MS3 reporter ion intensities were normalized using ProteiNorm [38]. The data were normalized using VSN [39] and analyzed using proteoDA to perform statistical analysis using Linear Models for Microarray Data (limma) with empirical Bayes (eBayes) smoothing to the standard errors [40]. A similar approach was used for differential analysis of the phosphopeptides, with the addition of a few steps. The phosphosites were filtered to retain only peptides with a localization probability > 75%, filter peptides with zero values, and log2 transformed. Limma was also used for differential analysis. Proteins and phosphopeptides with an FDR-adjusted *p*-value < 0.05 and an absolute fold change > 2 were considered significant and used for heatmap generation. Raw data are available in Supplementary Data (Proteomic Profile Data). The heatmap was generated using MetaboAnalyst5.0 software [41]. Data were normalized using pareto scaling and statistically compared using the T-test with the false discovery rate (FDR) calculated. Heatmap analysis with data clustering was performed using Euclidean distance measuring and a Ward clustering algorithm.

### 4.3. RNA Extraction and RT-qPCR

Cell pellets were collected, and flash-frozen using liquid nitrogen. Cell pellets were lysed with 350 μL RLT^®^ buffer, which has a proprietary composition (Qiagen, Hilden, Germany), followed by dissociation using Qiashredders tubes (Qiagen) and centrifuged at 12,500 rpm for 2 min. RNA was isolated using the RNeasy Mini Plus Kit (#74034) and the QIAcube instrument, both purchased from QIAGEN (Hilden, Germany), using the manufacturer’s protocols. Quantification of RNA was performed using a NanoDrop spectrophotometer (Thermo Fisher Scientific, Waltham, MA, USA). 

A quantity of 1 µg of cDNA was synthesized from the total RNA using a LunaScript^®^ RT SuperMix Kit (New England Biolabs #E3010L, Ipswich, MA, USA) per the manufacturer’s protocol. cDNA was diluted with nuclease-free water to achieve a final concentration of 10 ng/µL. 

A quantity of 2 µL of cDNA (20 ng) was used in a 20 µL qPCR reaction and analyzed using the BioRad CFX96 Touch Real-Time PCR Detection System (Hercules, CA, USA) and the Luna^®^ Universal qPCR Master Mix (New England Biolabs # M3003E). qPCR cycle conditions were one cycle of 2 min at 95 °C, 40 cycles of 5 s at 95 °C, and 30 s at the annealing temperature of 60 °C. Expression levels were determined from the values of threshold cycle (Ct) using the method of 2^–∆∆Ct^ and using RPLP0 or ACTB as the reference control genes. Primers were purchased from IDT (Coralville, IA, USA) and are listed in the Supplementary Data (Table S1).

### 4.4. ProteinSimple Protein Analysis

Simple Western blot was used to measure and analyze protein expression as previously described [42]. In brief, cells were washed twice with ice-chilled phosphate buffered saline followed by lysis in Radio-immunoassay Precipitation Assay (RIPA) lysis buffer supplemented with PMSF, protease inhibitor cocktail, and sodium orthovanadate (Santa Cruz Biotechnology, Dallas, TX, USA). The cell lysate was sonicated twice for 15 s each time and was centrifuged at 11,000× *g* for 15 min to remove cellular debris. Protein concentration was determined using the Pierce Bicinchoninic acid (BCA) protein assay kit (Thermo-Scientific Pierce, Waltham, MA, USA). Diluted protein lysates were combined with 5X fluorescent master mix (ProteinSimple, San Jose, CA, USA) that has dithiothreitol, fluorescent standards, and a system control protein (26 kDa) then was denatured by heating (95 °C for 5 min). Protein lysate was separated, and immunodetection of target proteins was performed using a capillary-based Jess Simple Western instrument (ProteinSimple, San Jose, CA, USA) using the manufacturer’s protocol. The ProteinSimple system control protein (26 kDa) served as an internal control and was also used to normalize protein expression (in the case of PSMB2 and PSMB5, B-actin was used as control because of the close molecular weight of these proteins and the system control). A quantity of 4 µL of each sample (0.5 µg/µL) was analyzed for target protein expression. The concentrations of protein lysate and antibodies/dilutions that were used are listed in Supplementary Data (Table S2). The uncropped blots are provided in Supplementary Data (Figure S1).

### 4.5. Proteasomal Inhibition with Bortezomib Cycle Treatment

Bortezomib treatment was performed in one cycle. During the treatment cycle, HRTPT cells were incubated four times (on the 1st, 4th, 8th, and 11th day) with a 1:1 mixture of DMFM: F12 medium containing 12.5 nM of bortezomib for 24 h. The treatment regimen within the cycle was designed to mimic the standard treatment of patients with multiple myeloma [43,44]. The medium was replaced with a bortezomib-free medium between each treatment. After the end of the treatment cycle, cell lysates were collected for RNA and protein, as described above.

### 4.6. Crystal Violet Assay

Cells seeded in 12-well or 6-well plates were treated with bortezomib for 48 h. Media were removed and 0.5% crystal violet in 20% methanol added to followed by gentle shaking at room temperature for 15 min, after which the plate was washed with water to remove excess crystal violet and left to air-dry overnight. Cells were lysed with 0.1 M sodium citrate in 25% ethanol (pH 4) and the absorbance was measured using a microplate spectrophotometer (BioTek EL800) at a wavelength of 595 nm.

### 4.7. Flow Cytometry Analysis

#### 4.7.1. CD133 CD24 Analysis

HRTPT cells were seeded in a 12-well plate at 250,000 cells/well and allowed to grow until confluency. Cells were fed fresh medium supplemented with bortezomib purchased from Selleckchem (#S1013, Houston, TX, USA) and cultured for 48 h at 37 °C and 5% CO_2_. Cells were washed with PBS twice and then detached using TrypLE enzyme (#12563029, Thermo Fisher Scientific) and centrifuged at 1200 rpm for 3 min. Cells were transferred to fresh Falcon™ tubes (#352235, Thermo Fisher Scientific) and resuspended in staining buffer (PBS + 5% fetal bovine serum). Cells were labeled with anti-human CD133 primary antibody conjugated to APC (1:20, #130-113-106, Miltenyi Biotec, Bergisch Gladbach, Germany) and anti-human CD24 primary antibody conjugated to FITC (1:20, #130-127-493, Bergisch Gladbach, Germany). Flow cytometry analysis was performed using a Sony SH800S cell sorter. The flow cytometry results were analyzed using FlowJo™ v10.8 Software (BD Life Sciences, Franklin Lakes, NJ, USA).

#### 4.7.2. Proteasomal Activity Probe Analysis

HRTPT cells were seeded in a 12-well plate at 200,000 cells/well and allowed to grow until confluency. Cells were fed fresh medium supplemented with 5 µM of the proteasomal activity probe (Me4BodipyFL-Ahx3Leu3VS) that was purchased from R&D systems (#I-190, Minneapolis, MN, USA), and cells were allowed to grow for additional six hours. Then the cells were washed with PBS twice, detached using TrypLE enzyme, and centrifuged at 1200 rpm for 5 min. Cells were transferred to a fresh Falcon™ tube and were resuspended in a staining buffer (PBS + 5% fetal bovine serum). Flow cytometry analysis was performed using a Sony SH-800 cell sorter. The flow cytometry results were analyzed using FlowJo™ v10.8 Software (BD Life Sciences).

### 4.8. Sphere Formation Assay

HRTPT cells were cultured in a T-25 flask and allowed to reach confluency. Cells were refreshed with a medium supplemented with either 12.5 nM bortezomib or Dimethyl sulfoxide (DMSO) as a control for 48 h, after which cells were detached with the TrypLE enzyme and 1000 cells/cm^2^ were seeded into a Corning™ T-25 ultra-low attachment flask (#07-200-876, Thermo Fisher Scientific) supplemented with 6 mL of DMEM: F12 serum-free medium with either 12.5 nM bortezomib or DMSO as a control. Cells were allowed to grow undisturbed for seven days at 37 °C in a 5% CO_2_ incubator. The spheres were harvested from the flask and transferred to a 15 mL tube. The flask was rinsed with 2 mL of DMEM/F12 and combined with the collected suspension, followed by centrifugation at 200× *g* for 5 min. The supernatant was carefully aspirated, ensuring the sphere pellet was not disturbed, followed by gentle resuspension to a final volume of 500 μL with DMEM/F-12. Then, 50 μL of the spheres suspension was added to each well of the 96-well plate, and the spheres were visualized and counted in each well under a microscope. Sphere formation efficiency was calculated by dividing the number of spheres counted by the number of seeded cells then multiplying by 100.

### 4.9. Matrigel™ Tubular Differentiation

The 96-well plate was coated with 40 µL of Okamatrix Matrigel (# 354230, OkaSciences, Kelowna, BC, Canada) and allowed to solidify for 30 min. HRTPT cells were seeded at 70,000 cells per well in 150 µL of serum-free DMEM: F12 culture media supplemented with 12.5 nM of bortezomib, and cells were allowed to attach for 24 h at 37 °C in an 5% CO_2_ incubator. The next day, 25 µL of Okamatrix Matrigel was added per well to stabilize the tubular formation. Cells were allowed to grow, and the medium was refreshed every 48 h. Light microscopy was used to follow tubular formation.

## Data Availability

Not applicable.

## Supplementary Materials

The following supporting information can be downloaded at: https://www.mdpi.com/article/10.3390/ijms241713303/s1.
