# Supplementary material for: Proteasomes Are Critical for Maintenance of CD133+CD24+ Kidney Progenitor Cells
Source: Int J Mol Sci. 2023 Aug 27;24(17):13303. doi: 10.3390/ijms241713303 (PMC10487892; doi:10.3390/ijms241713303)

<u>Supplementary Data</u>

Table S1: List of the used primers.

Primer	Catalog No./unique Assay ID	Company
CD133	Hs.PT.58.28257943	Integrated DNA Technologies
CD24	Hs.PT.58.45758278	Integrated DNA Technologies
RPLP0	Hs.PT.39a.22214824	Integrated DNA Technologies
ALDH1A1	Hs.PT.56a.38450309	Integrated DNA Technologies
PAX2	Hs.PT.58.40853724	Integrated DNA Technologies
KIM1	Hs.PT.58.3472	Integrated DNA Technologies
KRT8	Hs.PT.58.22681010	Integrated DNA Technologies
KRT18	Hs.PT.58.4200217	Integrated DNA Technologies
KRT19	Hs.PT.58.4188708	Integrated DNA Technologies
OCT4	Hs.PT.58.14648152	Integrated DNA Technologies
SOX2	qHsaCED0036871	BioRad
ACTB	qHsaCED0036269	BioRad
PSMA1	Hs.PT.58.4828216	Integrated DNA Technologies
PSMA6	Hs.PT.58.1535981	Integrated DNA Technologies
PSMB2	Hs.PT.58.40074074	Integrated DNA Technologies
PSMB5	Hs.PT.58.4649000	Integrated DNA Technologies
PSMB6	Hs.PT.58.19338999	Integrated DNA Technologies
PSMB7	Hs.PT.58.26457907	Integrated DNA Technologies
PSMC1	Hs.PT.58.4107601	Integrated DNA Technologies

PSMC4	Hs.PT.58.4728489	Integrated DNA Technologies
PSMD8	Hs.PT.58.4284519	Integrated DNA Technologies
PSMD9	Hs.PT.58.39005859	Integrated DNA Technologies
PSMD10	Hs.PT.58.15711631	Integrated DNA Technologies
PSMD11	Hs.PT.58.3899584	Integrated DNA Technologies
PSMG1	Hs.PT.58.4496078	Integrated DNA Technologies
PSMG2	Hs.PT.58.15536135	Integrated DNA Technologies
PSME1	Hs.PT.58.27483833	Integrated DNA Technologies
PSME2	Hs.PT.58.40021778	Integrated DNA Technologies

Table S2: List of the primary antibodies.

Primary Antibody	Supplier	Species	Dilution	Protein Conc
**CD133**	Cell Signaling #64326	Rabbit mAb	1:100	0.5µg/µl
**B-Actin**	Cell Signaling #8457	Rabbit mAb	1:400	0.5µg/µl
**PSMB2**	Santa Cruz # sc-58410	Mouse mAb	1:50	0.5µg/µl
**PSMB5**	Cell Signaling # 12919	Rabbit mAb	1:100	0.5µg/µl
**PSMD8**	Santa Cruz #sc-514053	Mouse mAb	1:50	0.5µg/µl

Figure S1: uncropped ProteinSimple blots for figures 2, 4, and 5 (A) PSMB5, (B) PSMB2, (C) PSMD8, (D) CD133 proteins dose-response to bortezomib 48hrs. (E) CD133 proteins cyclic bortezomib treatment on day 12.

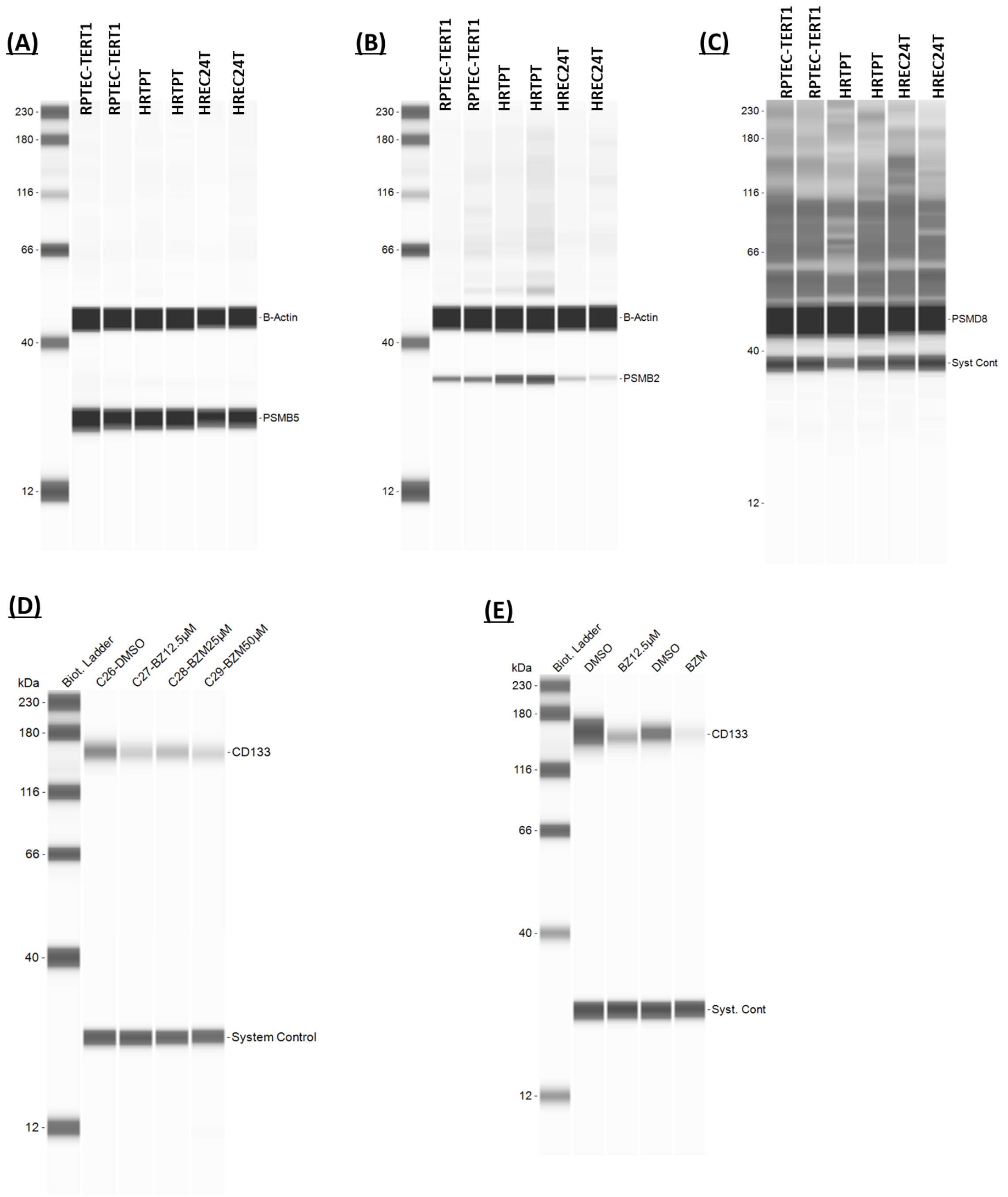

Figure S2: RT-qPCR analysis of mRNA levels of stem/progenitor markers ALDH1A1, PAX2, OCT4, SOX2, and kidney injury markers (KIM1, KRT8, KRT18, and KRT19) in HRTPT cells treated with bortezomib for 48 hours showing downregulation of stem/progenitor markers and the kidney injury markers that are markers of proximal tubular injury which excludes tubular injury due to bortezomib treatment even at high doses of 100 nM or 200 nM.

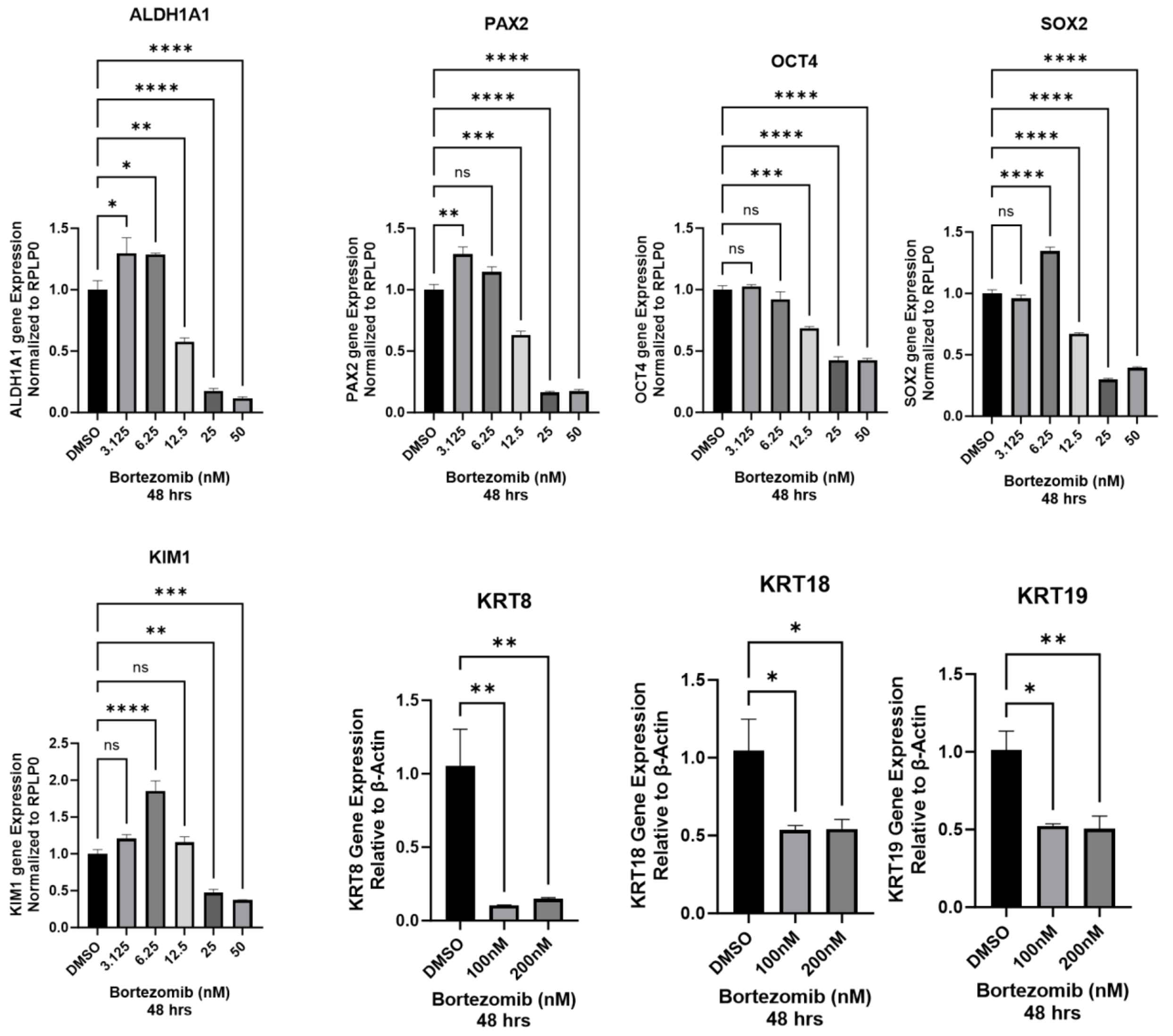

Figure S3: Light microscopy images of HRTPT cells treated with carfilzomib (0.5, 1, 5, 10, 20) µM for 48 hours (100x magnification).

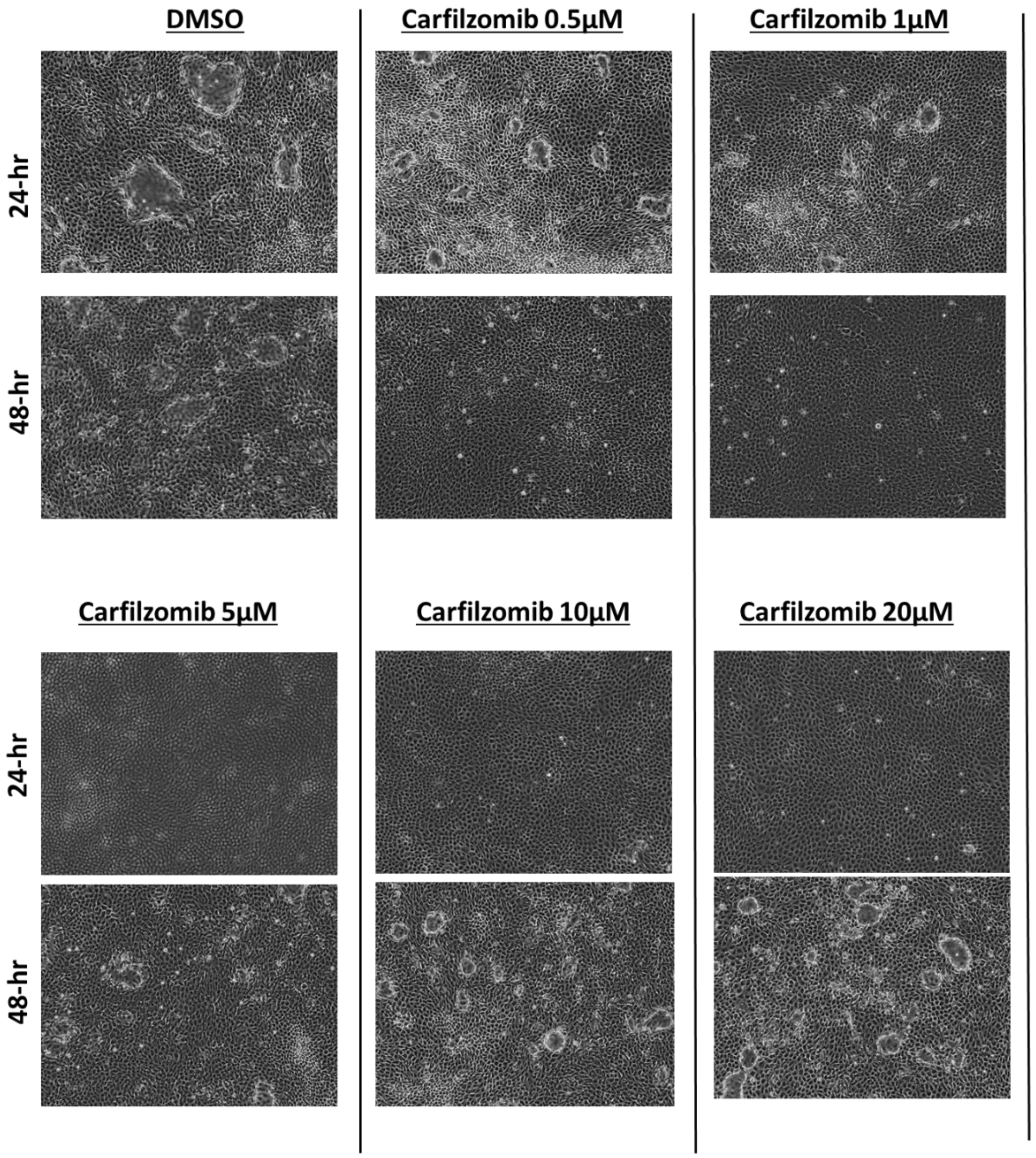

Supplement: Supplementary file 1 [file ijms-24-13303-s001.zip › Supplemental Figures S1.pdf]
